# Abundant Topological Outliers in Social Media Data and Their Effect on Spatial Analysis

**DOI:** 10.1371/journal.pone.0162360

**Published:** 2016-09-09

**Authors:** Rene Westerholt, Enrico Steiger, Bernd Resch, Alexander Zipf

**Affiliations:** 1 GIScience, Institute of Geography, Heidelberg University, Heidelberg, Germany; 2 Z_GIS, Department of Geoinformatics, University of Salzburg, Salzburg, Austria; 3 Center for Geographic Analysis, IQSS, Harvard University, Cambridge, MA, United States of America; Peking UIniversity, CHINA

## Abstract

Twitter and related social media feeds have become valuable data sources to many fields of research. Numerous researchers have thereby used social media posts for spatial analysis, since many of them contain explicit geographic locations. However, despite its widespread use within applied research, a thorough understanding of the underlying spatial characteristics of these data is still lacking. In this paper, we investigate how topological outliers influence the outcomes of spatial analyses of social media data. These outliers appear when different users contribute heterogeneous information about different phenomena simultaneously from similar locations. As a consequence, various messages representing different spatial phenomena are captured closely to each other, and are at risk to be falsely related in a spatial analysis. Our results reveal indications for corresponding spurious effects when analyzing Twitter data. Further, we show how the outliers distort the range of outcomes of spatial analysis methods. This has significant influence on the power of spatial inferential techniques, and, more generally, on the validity and interpretability of spatial analysis results. We further investigate how the issues caused by topological outliers are composed in detail. We unveil that multiple disturbing effects are acting simultaneously and that these are related to the geographic scales of the involved overlapping patterns. Our results show that at some scale configurations, the disturbances added through overlap are more severe than at others. Further, their behavior turns into a volatile and almost chaotic fluctuation when the scales of the involved patterns become too different. Overall, our results highlight the critical importance of thoroughly considering the specific characteristics of social media data when analyzing them spatially.

## Introduction

One aspect in the analysis of social phenomena is the search for spatial structures and patterns. The aim thereby is to explain the organization of complex spaces such as urban areas [1, 2] as well as social behavior patterns [3, 4]. Twitter and related social media feeds have recently become promising data sources in this regard. These online social networks capture a vast amount of georeferenced data from the everyday life of users, and are thus expected to represent a fraction of social happenings in geographic space. However, user-generated datasets have some unique shortcomings, such as their potential lack of trustworthiness, missing representativeness with respect to demographics, and self-selection bias [5]. The authors of [6] further describe this from a technical perspective by highlighting potential spatial, temporal and semantic inaccuracies. Nevertheless, Twitter provides a high temporal and spatial resolution, offering a unique opportunity to gain novel insights into the spatiotemporal behavior of humans.

A large body of literature dealing with social media analysis from geographic and social sciences has evolved throughout the last years. Examples span across a broad variety of fields such as the investigation of human mobility [7, 8], natural hazards and disaster management [9, 10], and geodemographics [11, 12]. These research efforts are summarized by [13] through providing a systematic literature review emphasizing spatial analyses of social media feeds. These authors note that one important but prevailing shortcoming is the naïve application of existing spatial methods when conducting social media analysis. Similar critiques including a lack of theory have recently also been raised elsewhere [14], though from a less technical perspective. Most established spatial methods were designed for datasets with different characteristics, i.e., data generated in some well-defined acquisition processes. It is therefore questionable whether existing methodological approaches produce reliable results. Although a majority of applied and empirical research on social media has been carried out, the scientific community is still lacking a thorough understanding of the interplay between applied spatial analysis methods and the specific characteristics that come with social media data.

One of the main differences between social media feeds and more traditional datasets is the data collection process, which appears highly unstructured. Mutually independent social media users contribute information about numerous real-world as well as fictional phenomena. To further stress the heterogeneity argument, issues arise even within representations of single phenomena. Due to varying spatial cognition and perception skills, user-generated data face the problem of user-induced heterogeneity (cf. [15] and [16]). Different phenomenon representations thus occur simultaneously, and their geometric overlap leads to a disrupted topology, whereby we refer to topology as the spatial arrangement of tweets. The result is a number of topological outliers that would not occur when only one phenomenon would be reflected in a clear manner in the data. Intuitively, the data acquisition process of social media thus causes spatial analysis methods to combine different actually unrelated tweets. Established density-based clustering techniques like DBSCAN [17], for instance, include tweets that represent different underlying phenomena. The result then is an averaged density being too high for some and too low for other reflected phenomena. Similarly, covariance-based techniques incorporating attribute values like Kriging [18] infer their spatial relationships from misleading tweet comparisons when incorporating different phenomenon representations. In all these cases, the analysis results might lead to wrong conclusions. Clearly, such techniques are designed for mono-categorical and spatially exclusive datasets. The following Section ‘A motivating example’ provides an example from a London twitter dataset indicating the abovementioned problem statement.

In this paper we investigate how topological outliers caused by the abovementioned heterogeneities influence spatial analysis methodology in a general sense. The problem outlined above is not restricted to any specific method, but prevalent across a range of spatial analysis techniques when applied on highly uncertain user-generated datasets. Therefore, instead of studying any specific spatial method, we rather investigate the underlying characteristic called spatial autocorrelation. This second-order data characteristic drives spatial patterning and is the conceptual basis for spatial analysis (see [19]). Analyzing spatial autocorrelation hence guarantees a high degree of generalizability of our results beyond the specificities of any particular technique. Table 1 lists all investigations that we conduct within this paper, including associated methodology. These tasks cover a broad range of issues around topological outliers and the way how these influence spatial analyses.

**Table 1:** Overview of the investigations conducted in this paper.

Scientific objectives		Methods
1)	Determination of the interplay between tweets and spatial analysis methodology. Illustration of unexpected behavior when spatially analyzing tweets.	Semivariogram, autocovariance
2)	Calculation and mapping of increased topological heterogeneity caused by pattern overlap. Demonstration of an additionally induced topological outlier region, which controls spatial patterning.	Eigenvalue analysis of local spatial weight matrices
3)	Influence of topological heterogeneity on the distribution of Moran’s ℐ (a measure of spatial autocorrelation). Determining consequences for drawing inference about spatial patterns.	Eigenvalue analysis of a global spatial weight matrix, violin plot
4)	Discovery of effects of topological outliers on spatial pattern quantification. Identification of disturbing spatial components induced by increased topological heterogeneity.	Moran’s ℐ, Moran scatterplots
5)	Determination of the role of scale differences between overlapping patterns on spatial analysis. Detection of regularity and chaotic behavior within the disturbing components from 4.	Serial correlation, correlograms

The remainder of this paper starts out with further motivating our research (Section ‘A motivating example’) and putting it into context (‘Spatial analysis and spatial heterogeneity’). We then outline some problematic covariation-based characteristics that emerge when analyzing Twitter messages with established spatial analysis methods (‘Indications from the Twitter dataset’). Afterwards, we investigate these characteristics within a simulated dataset, the latter allowing us to control different parameters such as spatial scale and attributes. We analyze how geometric overlap influences the power characteristics of conclusions drawn by spatial methods and how the topological arrangement pre-determines the range of expected results (‘Increased topological variability’). Afterwards, we identify interfering components that lead to spurious and misleading analysis results (Section ‘Influences on spatial autocorrelation’). This includes an analysis of their interdependencies with scale-differences among overlapping patterns (Section ‘The roles of scale differences and the degree of overlap’). The article closes with a discussion of the achieved results and concluding remarks, the latter including future research prospects and practical hints for scholars employing georeferenced social media data.

### A Motivating Example

**Fig 1:**
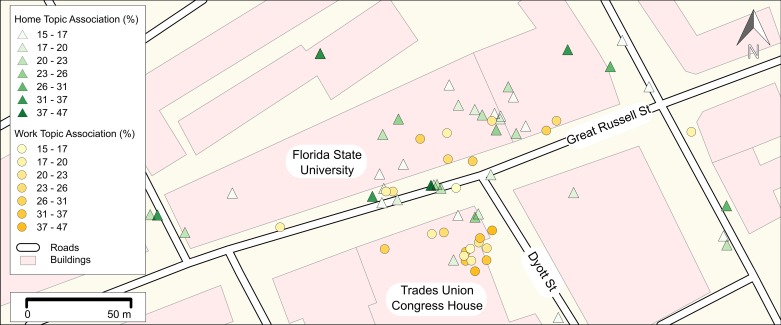
Map showing overlapping tweets in central London. The yellowish tweets represent a semantic “work” topic described in the following section. The greenish tweets, in contrast, were assigned a “home” topic (cf. [20] for details on these topics). The background map is based on OpenStreetMap data.

Fig 1 provides an example by showing geotagged tweets that occurred at the ‘Trades Union Congress House,’ an umbrella organization of British labor unions headquartered in London. The tweets and their attributes are drawn from another study that we conducted earlier (see Section ‘Datasets’ for an explanation, [20]). We analyzed the spatial pattern of work-related tweets by comparing them against the census workday population. For that purpose we carefully extracted latent topics. The colors in Fig 1 represent a semantic topic: either “home” (green) or “work” topics (yellow). Considering the work-related tweets, note the spatially overlapping variability within the yellow color-code. We can see that the spatial scales (i.e., the point-spacing) as well as the intensities of the topic assignments (i.e., the attribute values) fluctuate within small areas. This is an indicator for different phenomena or processes being reflected within tweets. It is likely that staff, as well as visitors and the general public, report about different work-related topics in this given area. Besides, the green home topic spatially coincides with the work topic in the northern parts of the observed region. This area is close to a university campus. Intuitively, students can be expected to tweet from this location. Some of their tweets deal with topics being classified as work-related phenomena (e.g., study-related topics), while some others are instead related to leisure activity (home-topic). This shows that both phenomena (home and work) as well as sub-processes of these (within-color variations) appear in a spatially overlapping manner. Thus, it can be concluded that social media datasets are of multi-categorical nature and spatially intertwined. This indicates an abundance of topological outliers as, unlike with non-overlapping patterns, their topology is highly diverse. They possess geometric characteristics from at least two different processes. Further, the overlap itself creates additional geometric characteristics. These outliers can be expected to influence the outcomes of spatial analyses and are the starting point of this paper.

### Spatial Analysis and Spatial Heterogeneity

We should first briefly articulate our problem statement in terms of traditional concepts of the field of spatial analysis. The overlap of phenomena, to which we are referring, manifests itself as a specific type of spatial heterogeneity. Spatial heterogeneity traditionally refers to a variable’s response to extrinsic spatially varying environmental or socio-economic conditions. This typically leads to varying intensities, which in turn designates spatial heterogeneity as a first-order effect (in contrast to the second-order effect of spatial dependence) [21]. Common forms of spatial heterogeneity are ‘spatial regimes’ (patchy areas of varying intensity, abrupt changes) and ‘trends’ (smooth transitions between means) [22]. Regimes are common in urban areas and typically resemble the local-scale variability of such regions [23], while trends are more important to the physical sciences [24]. Dealing with these kinds of spatial heterogeneity is a widely discussed topic in spatial research. It is methodologically reflected by a range of methods such as local measures of spatial dependencies [25, 26, 27], separate treatments of different regimes [28, 29, 30], approaches for determining the local scales of patches [31, 32] and local regression models like ‘geographically-weighted regression’ [33, 34], ‘spatial expansion’ [35, 36] and a localized version of ‘spatial eigenvector filtering’ [37].

All approaches mentioned above assume that spatially exclusive forms of heterogeneity are observed. That is, they refer to one of the traditional types of spatial data: geostatistical data (spatially continuous phenomena), lattice data (spatially discrete phenomena) or event data (stochastic geometries) [38]. Event data incorporates superposition of phenomena to a certain degree, but, however, falls back to a lattice when analyzing attributes (different types of the ‘mark correlation function,’ cf. [39]). These spatial data types are reasonable with many kinds of spatial data such as census or housing data. The outlined Twitter example from the previous section, however, shows that social media data typically violate the assumption of spatial exclusiveness and cannot be straightforwardly assigned to one of the data types mentioned above. That is, with respect to spatial heterogeneity, social media data show a novel kind of that characteristic. Spatial heterogeneity here is caused by the unstructured data acquisition process (i.e., an extrinsic source) and is characterized by the superposition of phenomena. It expresses itself by the formation of specific (artificial) regimes within the ‘zones of overlap.’ These zones appear where different phenomena or processes coincide within data and show abnormal behavior with respect to statistical and topological characteristics. Just as with traditional forms of spatial heterogeneity, this effect is likely to influence the outcomes of spatial analysis, eventually leading to spurious results. These zones of overlap are what we target by our research.

## Materials and Methods

### Ethics Statement

Some of the data used within this study was crawled from the microblogging service Twitter. We have eliminated all references to actual Twitter users. Therefore, the dataset is anonymized and does not violate the privacy of actual persons.

### Datasets

We use two different datasets for our analyses. One of them is a Twitter dataset consisting of georeferenced tweets. It has been crawled through the publicly available Streaming API during a period of approximately one year. The sample used here is an excerpt of a much larger dataset consisting of 20 Million tweets covering Greater London, which was used in one of our previous studies [20]. We only leveraged explicit coordinates offered in the form of latitude-longitude tuples. This may include GPS-derived locations as well positions determined by WiFi-positioning techniques and check-ins (see Section ‘Indications from the Twitter dataset’ for further discussion of this point). That is, we did not include location tags like “London, UK.” The latter would blur up the analysis scale as these do not refer to points but to much larger polygons instead. Our pre-processing includes several natural language processing steps such as tokenization, stop word removal and stemming. Through these steps we remove a great deal of potentially unnecessary noise that might disturb the analysis if not being eliminated. What we did not remove is artifacts such as tweets contributed by bots. Removing these is still an issue of ongoing research (e.g., [40], [41] and [42]). Further, we do believe that, through our semantic treatment (see below), a lot of these artificial tweets have been removed implicitly. The semantic modelling was done by means of Latent Dirichlet Allocation (LDA) [43], a probabilistic bag-of-words model for extracting latent topics from text corpuses. Please refer to the paper mentioned above for a more detailed explanation of all our conducted pre-processing as well as semantic processing steps. After preprocessing and narrowing down the scope to one latent topic (“work”), approximately 23,000 tweets remain. The attribute used here is a percentage expressing the degree of tweet-topic association. The chosen topic “work” represents a range of business activities and personal reports about individual daily commute, day-to-day work experiences and similar phenomena.

**Fig 2:**
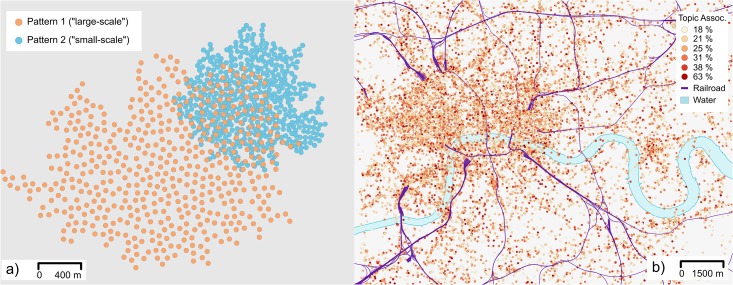
Overview of the two employed datasets. a) Simulated pattern, colors indicate the two primal sub-pattern. b) Twitter data from London. The background map of b) is based on OpenStreetMap data.

The second dataset used in this paper is a simulated point pattern. It resembles an overlap of two different spatial processes reflected within social media. Attributes attached to the points were drawn from Gaussians. In an initial configuration, these center on different levels of intensity (*μ*_1_ = 250, *μ*_2_ = 750) while possessing a similar variance ($\sigma_{1}^{2}=\sigma_{2}^{2}=22,500$). Each of the involved sub-patterns shows spatial autocorrelation of 0.81 (Moran’s ℐ, IDW-based spatial weights). They do therefore mimic positive spatial autocorrelation and first-order spatial heterogeneity as it is described in Section ‘Spatial Analysis and Spatial Heterogeneity.’ Both involved sub-patterns operate at different spatial scales, whereby scale is defined in terms of point spacing within this study. The smaller-scale process operates at an interval of [40*m*, 50*m*], whereas the larger-scale process interacts at distances on [70*m*, 80*m*]. The geometries of the patterns were generated by a random walk approach. An initial point was placed arbitrarily. Then, starting from that point, 500 points were successively placed by choosing a random angle and distance at each step, both of which are following a uniform distribution constrained by the abovementioned distance intervals. In total, 1,000 points were placed. Now, by overlaying these two patterns, we simulate an overlap as observed within the motivating example above. The degree of overlap has been chosen such that 23.8% of the points from the large-scale pattern interact with at least one point from the small-scale counterpart. This idealized dataset allows us to vary the scales of the involved sub-patterns in an archetypical way and allows controlling the attached attribute values. It thus allows isolating and investigating different topological effects of sampling-induced spatial heterogeneity on outcomes of spatial analysis while avoiding any cultural, socio-demographic, topographic and other kinds of extrinsic influences (e.g., the bots mentioned earlier) that might bias the analysis outcomes. This guarantees a high level of generalizability of the achieved results. Fig 2 provides an overview of both datasets.

### Methods

#### Heat Map of Autocovariance Terms and Variographic Analysis

In a first step, we highlight the problem statement by means of the Twitter dataset. To achieve that we apply two different statistical measures to our Twitter data: sample autocovariance and semivariogram estimation. Sample autocovariance describes the degree of conformity over the mean across the realized tuples of topic associations. In its spatially unweighted form, the pairwise autocovariance matrix is defined as

$$s_{x,x}=\frac{1}{n}\cdot \left[\begin{array}{ccc} {\left(x_{1}-\bar{x}\right)}^{2} & \ldots & \left(x_{1}-\bar{x}\right)\left(x_{n}-\bar{x}\right)\\ \vdots & \ddots & \vdots \\ \left(x_{n}-\bar{x}\right)\left(x_{1}-\bar{x}\right) & \ldots & {\left(x_{n}-\bar{x}\right)}^{2} \end{array}\right]$$ (1)

where x_i_ and x_j_, in our case, denote two topic associations indexed over tweets i and j, and $\bar{\text{x}}$ is their corresponding mean. We investigate the off-diagonal elements from Eq 1 by relating them to their geographic distances measured between i and j. The result is a heat map of autocovariance mapped against distance. This heat map allows disaggregating the overall autocovariance into its constituting parts. The benefit of this approach is that, other than with a covariogram or a correlogram, we are neither aggregating by distance bands nor by random variables. We thus get a detailed picture of all available pairs of observations within their geographic context. Therefore, these pairwise comparisons reveal local information through geographic space.

We complement the abovementioned local viewpoint by a global summarization of spatial relations. This is done through constructing an empirical semivariogram. Let $p_{i}\in {\mathbb{R}}^{2}$ be geometric points (i.e., tweets) over which the topic associations x_i_ are spatially indexed. The empirical semivariogram is then estimated by [44]

$$\gamma \left(h\right)=\frac{1}{2N\left(h\right)}\cdot \Sigma_{i,j}^{N\left(h\right)}{\left(x_{i}-x_{j}\right)}^{2},\;\forall \left(x_{i},x_{j}\right)\;:\;\left|\left|p_{i}-p_{j}\right|\right|\in \left(h_{min},h_{max}\right)$$ (2)

where h_min_ and h_max_ span non-overlapping distance classes h, N(h) describes the numbers of pairs of points falling into these classes and ||⋅|| denotes the Euclidean distance measure. A semivariogram thus describes the variance within distance classes h. Our employed distance classes have a width of 25 m. This ensures a fine granularity and acknowledges the large numbers of tweets in dense packing.

Both these measures, autocovariance and semivariogram, are helpful devices for demonstrating the problem statement mentioned in the introduction. We use them to reinforce the issues underlying our research and to show indications for spatial overlaps within Twitter. While the semivariogram comes up with a well-understood interpretation allowing to demonstrate the unexpected behavior caused by overlaps, the heat map allows for explaining this behavior in greater detail by uncovering the types of interactions across space.

#### Moran’s ℐ and Moran Scatterplot

We are interested in analyzing general behavior beyond any specific spatial methods. The universal force underlying spatial methods is called *spatial autocorrelation*, which quantifies how strongly observations relate with each other in space and how this drives patterns [19]. Roughly put, spatial autocorrelation refers to “the coincidence of value similarity with locational similarity” ([45], p.241). Our simulation experiments are therefore based on Moran’s ℐ, the quasi-standard measure of spatial autocorrelation. Moran’s ℐ can be roughly characterized as a spatialized version of Pearson r, restricted to observations of a single random variable. Its equation is given by [46]

$$I=\frac{n}{\sum_{i,j}^{n}w_{ij}}\cdot \frac{\sum_{i,j}^{n}w_{ij}\left(x_{i}-\bar{x}\right)\left(x_{j}-\bar{x}\right)}{\sum_{i}^{n}{\left(x_{i}-\bar{x}\right)}^{2}}$$ (3)

where n denotes the overall number of observations. The factors w_ij_ denote elements of a spatial weight matrix. This matrix captures the geographic layout of the study area and defines neighborhood relations. It describes how much resistance the geographic topology bears upon the covariation within the associated attribute. We use an inverse-distance notion for our investigations, because our simulated data was created by underlying distance theory (geometric interaction ranges, see previous section). Clearly, in any empirical studies, the choice of weights is a crucial one and should be undertaken with care and expert knowledge of the underlying phenomenon. Other common weight choices are summarized by [47]. Investigating how topological social media characteristics influence Moran’s ℐ will allow us to make more general statements about its influence on spatial methods in a broader sense.

The Moran scatterplot is a graphical device complementing Moran’s ℐ. It was introduced by Luc Anselin [48] and provides a means to disaggregate spatial autocorrelation into its distinctive parts. Thereby, the regression line through this scatterplot is coincidental with the non-normalized Moran’s ℐ measure. Note that normalizing over spatial weights is not necessary here, since we do not vary the spatial layout during our study. Thus, analyzing the regression line in the Moran scatterplot is tantamount to analyzing Moran’s ℐ. For that reason, and because the graphical interpretation allows determining sub-components of spatial autocorrelation in greater detail, we use the Moran scatterplot for analyzing systematic disturbances to the spatial pattern caused by topological outliers.

#### Local Eigenvalues

Analyzing the topological configuration requires a measure of overlap and topological heterogeneity. For this purpose, we divide the spatial weight matrix from Moran’s ℐ into local submatrices and calculate their principal eigenvalues. The principal eigenvalues of these localized submatrices represent the local interaction potential attached to each single observation. The higher the eigenvalue, the higher is the variability within the geographic connectivity and thus the contribution of a single spatial unit to the entire region. In turn, high variability within pairwise connectivity relations means that a homogeneous pattern is disrupted by an overlap with another, eventually differently scaled, pattern. The eigenvalues thus summarize the overall degree of overlap of observations within their local geographic context as well as the strength of their influence contributed to spatial pattern assessments. We can calculate the spectra of local eigenvalues for the local matrices as [49]

$$\left\{-\sqrt{\Sigma_{j=1}^{n}w_{ij}^{2}},\ldots ,0,\ldots ,\sqrt{\Sigma_{j=1}^{n}w_{ij}^{2}}\right\}\cdot$$ (4)

Only the principal eigenvalues are non-zero. The importance of these eigenvalues for our investigations is that we can use them for summarizing local topological effects. They hence allow us to measure the intensity of geometric overlap of sub-patterns because an overlap is expected to produce outlier eigenvalues. We use this measure for analyzing the topological influences of overlap on the outcomes of spatial analyses.

Similarly, we also calculate the eigenvalues of the overall global spatial weight matrix. This matrix comes up with n non-zero eigenvalues. Again, the principal eigenvalues are of importance, because they determine the feasible range and the shape of the distribution of Moran’s ℐ values [49, 50]. The eigenvalues do hence predetermine the efficiency and power of Moran’s ℐ as a test statistic. This reinforces how crucial topological outliers are towards spatial pattern assessment and demonstrates why we use them as a useful proxy for topological heterogeneity.

#### Serial Correlation and Correlogram

The eigenvalues explained above capture the geometric and topological influences that single spatial units exert onto the entire region. Combining these with Moran’s ℐ allows analyzing how these (and especially topological outliers) affect the detection of pattern within attributes. This involves investigating how different parts of an overlapping pattern behave with respect to increasing scale differences of the involved overlapping pattern. We are interested in the coherence of these effects. Only when the behavior is somehow tractable, analysts can try to deal these issues. Chaotic behavior, in turn, would be hardly treatable. Thus, we estimate the serial correlation of the slope of disturbing components from the Moran scatterplot by means of the one-dimensional sample autocorrelation coefficient:

$$r\left(\tau \right)=\frac{\sum_{i}^{n-\tau }\left(x_{i}-\bar{x}\right)\left(x_{i+\tau }-\bar{x}\right)}{\sum_{i}^{n}{\left(x_{i}-\bar{x}\right)}^{2}}$$ (5)

where τ is the lag (here: the lag of scale differences in meters). We plot these estimates against the lag, which is then called a correlogram. This allows investigating the behavior of the overlap of patterns through scale differences.

## Results

### Indications from the Twitter Dataset

**Fig 3:**
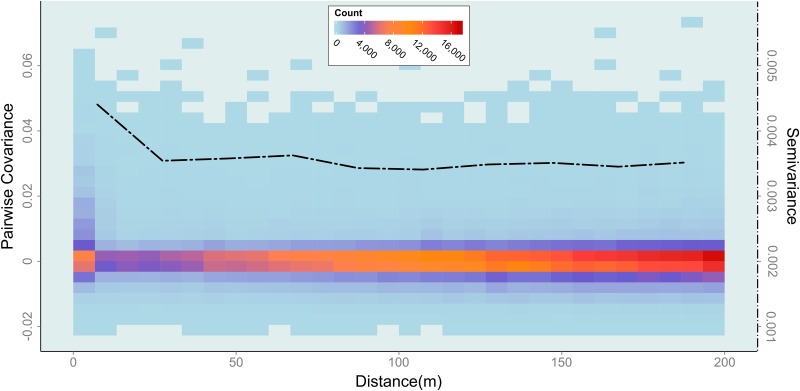
Heat map of pairwise covariance terms and semivariogram of topic associations. The dashed semivariogram refers to the right-hand y-axis (same line-style). The left-hand y-axis refers to the color-coded bins. Figure bases on the entire Twitter dataset from London, see Section ‘Datasets.’

Before conducting simulation experiments, we should turn our attention toward the Twitter dataset to highlight indications for geometric overlap of different phenomena. Fig 3 visualizes two kinds of information: a heat map of all pair-wise entries from the covariance matrix plotted against geographic distance (underlying color-coded bins) and a semivariogram (dashed line atop).

Observe the unusual course of the semivariogram. A typical semivariogram for mono-categorical datasets is of inclining nature when spatial effects are present. The attribute values are typically expected to be most similar in close geographic proximity. Thus, with increasing distance, the variability increases until the so-called ‘sill’ is reached. As of that point (called ‘range’) the variability levels off to the overall variance and is no longer assumed to be affected by geographic effects. The semivariogram shown in Fig 3, however, indicates a different behavior. It starts out at a high level of variation, and then progresses towards a constant level. That means that values are dissimilar when they are close to each other. At a first glance this indicates global negative spatial autocorrelation at short-ranges. The topic associations thus seem to possess some kind of repulsion behavior at a local scale. This kind of association, however, is rarely observed in real-world datasets [51]. We should thus further examine this distinctive behavior.

The underlying heat map within Fig 3 offers further insight to the notable behavior of the semivariogram. A significant accumulation of orange bins is observable at distances close to zero. These indicate a large fraction of mutually unrelated tweets at very local scales. Mutually unrelated tweets, however, do not hint on repulsion. They rather show that a great number of neighbored tweets are not related to each other at all, being neither systematically similar nor dissimilar. Apart from that, we can also find some indications for repulsion. Notice the small peak of blueish bins reaching downwards into the negatives. The latter partially supports the observed hint suggested by the semivariogram: we do indeed observe some opposed tweets in close vicinity. Besides these two findings, another important observation from Fig 3 is the high-reaching peak towards higher positive covariation. This peak indicates tweets that are similar to each other, and thus indicate clustering behavior caused by systematic spatial phenomena. These latter tweets are the ones we are typically interested in when searching for spatial pattern within social phenomena and processes. They hint on common behavior and thus (in the present case) semantically coherent spatial regions. It is further important to note that the heat map disaggregates the semivariogram. The semivariogram is based on combining multiple tweets, regardless of their underlying phenomena. This is exactly the problem with spatial methodology which we outlined in the introduction. The heat map, in contrast, reveals the underlying causes that ultimately lead to this issue.

Note that Fig 3 is based on all tweets that are found to be semantically associated with the topic “work.” This includes some spatially coincident tweets, which might be due to the Twitter data collection process (e.g., WiFi positioning techniques, Foursquare check-in data). For some kinds of investigations one might want to keep these duplicates (e.g., when characterizing places), but in some other cases one might wish to exclude them previously. S4 Fig in the appendix shows that, however, removing these spatial duplicates does not affect the general argumentation outlined above. It only diminishes the magnitude of spatial effects in short distance ranges. The latter is as expected, because removing spatial duplicates is essentially a modification of extremely local tweets. Other than Fig 3, S4 Fig is designed in a relative fashion for the sake of comparability (the numbers of tweets are changed after the removal process, absolute numbers are thus less effective).

In the remainder of this article we will use the controlled simulated dataset to further investigate the consequences of such overlap on different topological aspects of tweets. This allows isolating and controlling precisely the effects we are looking for.

### Increased Topological Variability

**Fig 4:**
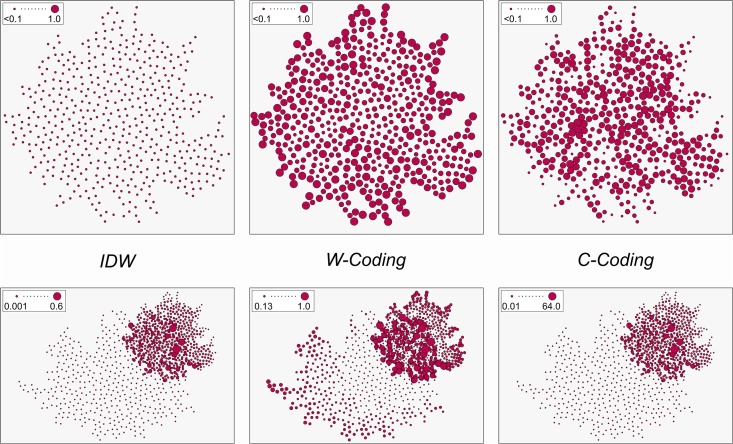
Local eigenvalues of a single pattern (top) and a combined pattern (bottom). Please note the differing value ranges, which are tributes to different distributions of eigenvalues across the maps. Size classification is Jenks natural breaks.

Whenever a single pattern is observed, all points interact at only one or rather few specific scales. In the best case the observed scales match those of the underlying phenomenon. Be it one or multiple scales, the crucial point is that these reflect the underlying causative phenomenon. In such cases, the variability within the relative spatial arrangement of points is relatively low and homogenous. Most points interact at similar or at least meaningful distances. When patterns overlap, however, points from different patterns are positioned in close proximity to each other. These different patterns might possess different kinds of point spacing characteristics. Thus, the topological diversity is higher and the number of cross-pattern interactions between actually unrelated points increases. This topological diversity is expressed by increased local eigenvalues of the spatial weight matrix as shown by Fig 4 (bottom). We should thus analyze how overlap of patterns influences these local eigenvalues.

The top row of Fig 4 shows eigenvalues for a single *non-overlapping pattern*. The left-most map thereby provides eigenvalues for the respective plain pattern. The two maps at the right-hand side demonstrate the effect of two different kinds of spatial weights normalizations (C and W-coding). These normalizations are often applied for making different spatial weight configurations comparable among each other (see [52] for an overview). It is well-known that, with non-overlapping patterns, such normalizations lead to topological outliers [49]. Indeed, we can see that W-coding emphasizes the boundaries of the pattern, while C-coding exaggerates its interior. The corresponding outlier observations show a strongly increased variability. Given that the normalization procedures are researcher-induced artifacts, such geographic layouts allow the corresponding outlier units too much interaction with their neighbors. This disrupts subsequent spatial analyses. The plain pattern, however, appears homogeneous. These results confirm previous research [49]. Moreover, note the generally low intensity of the eigenvalues in case of the plain pattern. None of the values exceeds 0.1. This shows that only one coherent underlying phenomenon is represented by the pattern as the topological heterogeneity is kept fairly low.

The bottom row of Fig 4 shows eigenvalues for an *overlapping pattern*. Again, the normalized patterns show similar tendencies as their non-overlapping counterparts. However, some differences are noticeable: First, the ranges of the eigenvalues reach up to a way higher intensity, especially with the C-coded pattern where the upper bound reaches up to a value of 64. This demonstrates that geometric overlap does not just produce outliers, but also seems to interact differently with different kinds of normalization techniques. Second, we can observe that, in contrast to the non-overlapping pattern, even the plain pattern now shows a large number of outliers. The topological variability is thus already increased by the mere fact that different patterns overlap and without having applied any normalization. The spatial neighborhoods of such points are composed of different scales simultaneously, making it difficult to adjust any proper analysis scale. The implication of these results is that, when analyzing social media data, it is highly likely to observe numerous such outliers. Moreover, overlap may place some of the points in very close proximity at distances shorter than one distance unit. This becomes a severe problem whenever geographic relationships are modeled by means of distance decay functions. Distance decay possesses abnormal behavior when distances are below one distance unit. Densely covered zones of overlap may thus yield extreme outliers in case of pattern overlap when using distance-based specifications of spatial interactions. This is reflected by Table 2, which provides Moran’s ℐ values for both kinds of patterns from above.

**Table 2:** Moran’s ℐ values under different weight specifications. W and C refer to row- and global normalization.

	IDW	Binary	IDW (W)	IDW (C)	Binary (W)	Binary (C)
**Single Pattern**	0.81	0.81	0.85	0.81	0.85	0.81
**Combined Pattern**	-1.07	0.42	0.42	-1.07	0.55	0.42

In order to compare the distance-based weights that were used above toward non-distance weights, we additionally included a binary weighting scheme. Thereby, the upper bounds of the respective point interaction ranges were used as cut-off distances. The attached attribute values are Gaussian as described in Section ‘Datasets.’ Again, different normalizations were applied (i.e., W and C). We see that the variation across different spatial weighting schemes is relatively low for the non-overlapping pattern (top row). As the points used here are placed relatively regular, this is in accordance to results obtained by [53], who investigated the impact of different weight configurations with regular raster-like patterns.

In contrast, the bottom row outlines results for the combined overlapping pattern. These show marked differences between the weight configurations. Both employed binary schemes behave relatively similar. Contrary, the distance-based weights indicate negative spatial autocorrelation. Recall that both involved patterns are actually positively autocorrelated through space. Thus, the extreme exaggeration of very close but very different points possesses a huge influence on the overall result, ultimately leading to a wrong conclusion about the spatial effects within the pattern. These results show how sensitive the topological outliers react on the type of weights in case of overlapping patterns. Table 2 underpins the importance of a careful choice of weights when analyzing geometrically overlapping social media data.

The results from above are also of importance to inferential statistics. Many global spatial statistics like Moran’s ℐ are defined in an averaging notion. They are defined as a weighted average of local counterparts (in this case: local Moran’s ℐ). This characteristic holds for all statistics and measures of the so called LISA type [25]. With these methods, single outliers control global statistics and influence their distributions. As we have seen above, these outliers are abundant within overlapping patterns. Thus, due to their increased abundance, these cause the probability of extremely high or low degrees of spatial association to increase artificially [50]. Whether high or low values are affected thereby depends on the type of outliers observed. The latter point flaws significance procedures and leads to wrong conclusions about spatial effects.

**Fig 5:**
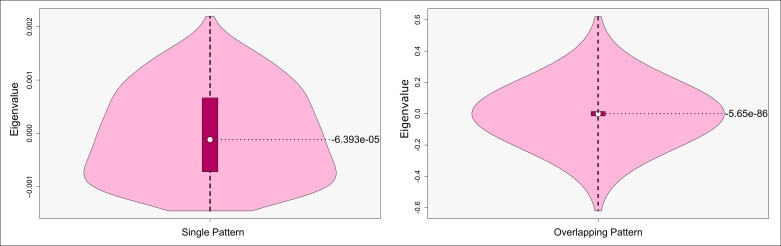
Violin plots (cf. [55]) for a single pattern (left) and an overlapping pattern (right). The central box illustrates data between first and third quartile. The white dot refers to the median.

The investigations above are based on *local eigenvalues*, which were in turn calculated from local submatrices. Combining these reveals the overall global spatial weight matrix, and, consequently, the respective *global eigenvalues*. These are of importance for the detection of spatial effects, because they reveal the shape as well as the range of the corresponding reference distribution of analysis outcomes [54]. In other words: the geographic layout determines the bounds of the strength of detectable effects. Fig 5 visualizes violin plots of these global eigenvalues for the two patterns analyzed above. The non-overlapping pattern shows a compact distribution. Half of the values including the median accumulate around the expectation of Moran’s ℐ, which is -0.001 in this particular case. Only few values extend to the extremes. These further span a distinctively narrow overall range. This means that any spatial test statistic which is evaluated on this geographic layout possesses favorable power and efficiency characteristics, as the range of possible outcomes is kept reasonably small. Thus, the measured strength of spatial effects has little room for fluctuating toward unrealistic erroneous choices. In case of the combined pattern we do also observe most of the values around the expectation of Moran’s ℐ. This time, however, the markedly broadened range along the y-axis shows that the range has been stretched towards a multiple of the previous one. This demonstrates how strong the outliers caused by the overlap worsen the power as well as the efficiency of spatial test statistics obtained from overlapping patterns.

### Influences on Spatial Autocorrelation

**Fig 6:**
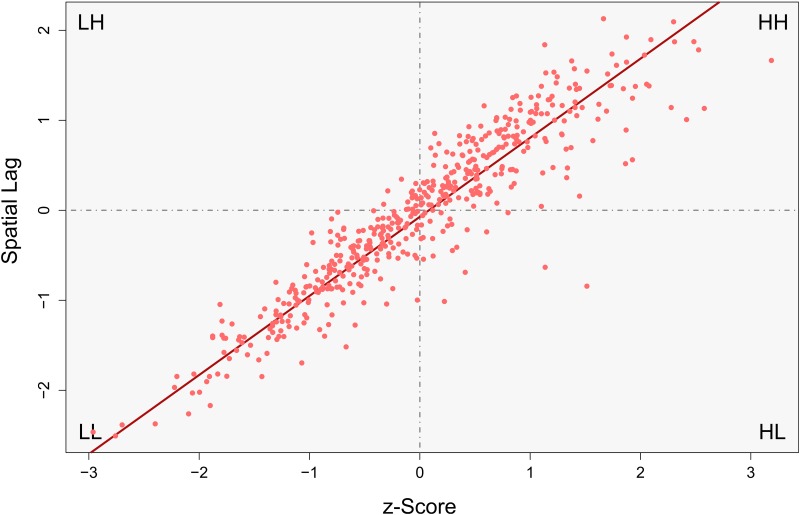
Typical Moran scatterplot for positively spatially autocorrelated data. Blue line shows the trend. HL: High-Low, LH: Low-High, LL: Low-Low and HH: High-High interaction.

A naturally arising question now is to ask for the specific consequences of the findings from above on spatial analysis. The analysis of topological variability conducted above is concerned with the geographic layout. However, attribute values were not yet included. A simple yet powerful tool to inspect the strength and type of spatial associations within attributes is the so called Moran scatterplot [48], which enables us to investigate how topological variability influences spatial analyses. Fig 6 showcases a Moran scatterplot for the non-overlapping pattern that was used in the previous section. A supplementary map of the underlying attribute values as well as a corresponding histogram is found in S1 Fig. The trend line through these points stretches from the third quadrant into the first one. This is the typical behavior in case of positive spatial autocorrelation. It indicates that most points are placed in geographic neighborhoods that consist of similar points. The first quadrant thereby means that high values are spatially surrounded by other high values (HH), while the third quadrant refers to low-low neighborhoods respectively (LL).

**Fig 7:**
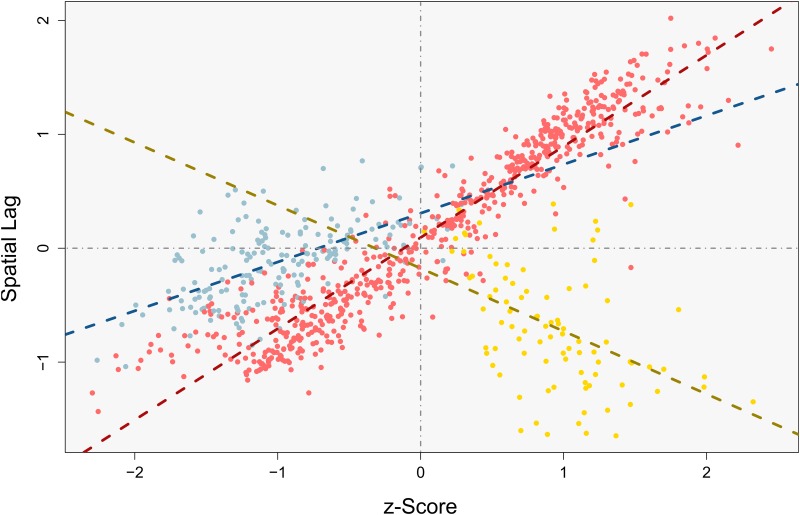
Moran scatterplot for the combined pattern. Dashed lines show the trends of the similarly colored points.

When we construct the same scatterplot for the overlapping pattern we see that a number of additional components appear within the plot (Fig 7, map and histogram in S2 Fig). The red points are observations which are unaffected by pattern overlap, and thus do not interact in a cross-pattern manner. The blue points belong to the smaller-scale process but do interact with points from the larger-scale one. In turn, yellow points are part of the larger-scale process but interact with the small-scale pattern. The corresponding lines demonstrate the respective trends for those three point clouds. Observe that the small-scale points from the overlapping area add a positive component which is paralleling the red points. The trend of this component, however, appears flatter than that of the red one. This means that, while still being positive, the blue points weaken the strength of observable spatial effects as they pull down the overall trend. Being even more influential, the yellow trend line shows negative behavior. The underlying points must therefore be negatively correlated with their spatial surrounding. Both these components together obscure the real pattern which is encompassed within the data. The actually searched pattern is inflated with numerous artificial interactions.

**Fig 8:**
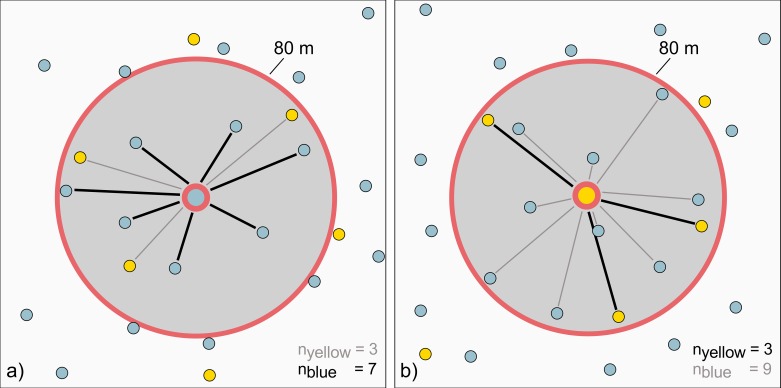
Interrelationships between points within the zone of overlap. a) from a small-scale perspective and b) from a large-scale perspective.

Why does the blue component add a positive trend, while the yellow component contributes a negative relationship? Fig 8 partly answers this question for the situation from Fig 7 by showing a magnified detail view from within the zone of overlap. We see that, in case of the small-scale points (8a), the number of interactions with similar points (i.e., other small-scale points) is still high. That is, although some yellow points are included, the majority of interactions still take place with other blue points. The yellow points are less frequent, because their scale, and therefore their point spacing, is lower. The few cross-pattern interactions between blue and yellow, however, are not without effect. They cause the blue component to be flatter than the red one. In contrast, Fig 8b shows the same situation from a yellow component perspective. Yellow points do interact frequently with blue ones within the zone of overlap. Since the latter operate at a different attribute value intensity (i.e., their attribute mean is higher), these interactions in close proximity appear as repulsion behavior. Repulsion, in turn, is indicated by negative spatial autocorrelation. This explains why the yellow component runs downwards yet forming a negative relationship.

**Fig 9:**
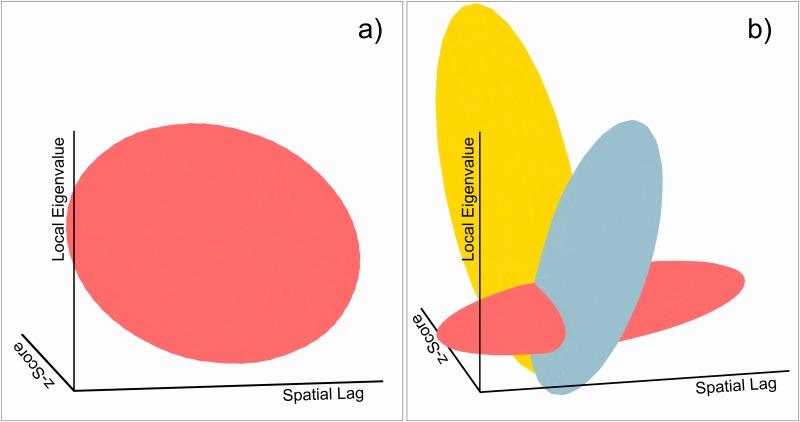
Comparison between the Moran scatterplot and associated local eigenvalues. Colors are in accordance to Fig 7. Shown ellipses mark the respective 95% confidence ellipses. a) Ellipse for a non-overlapping pattern. b) Ellipse for an overlapping pattern. Note that the magnitudes of the axes differ. Similar sizes were chosen for visualization purposes.

Fig 7 has shown two different disturbing components and Fig 8 shows that these are caused by different underlying topological constellations. In order to relate these components to topological variation, we relate them to their associated local eigenvalues. Fig 9 shows corresponding 3D plots relating the Moran scatterplots from above to their associated local eigenvalues. In case of a non-overlapping pattern (9a), the 95% ellipse appears slightly negatively correlated with the eigenvalues. Thus, as demonstrated above, the behavior is homogeneous. Fig 9b reveals that the disturbing components react differently on the degree of overlap. While the yellow component possesses a negative relationship with the eigenvalues, the blue component tends towards a positive connection with increasing degrees of overlap. This supports our conclusions drawn from Fig 8, because both these effects seem to become stronger with increasing local eigenvalues. Overall, the plot shows a distinctive shape. It reveals that different parts of the overlapping pattern react in different ways on the topological implications that come along with the overlap.

### The Role of Scale Differences

The results from above unveil that geometric overlap influences the quantification of patterns. We now turn our attention to effects that govern these influences, namely the effects caused by scale differences between the involved patterns. We investigate this by means of testing a range of scale differences between the involved overlapping patterns.

#### Influence of Scale Differences on the Numbers of Interactions

All previously stated results are based on analyzing a single combined pattern. Yet, we don’t know how differing scales of the involved sub-patterns become effective. When social media patterns overlap, they can interact in two different general ways. One of these is a true geometric overlap. That is, a part of one pattern might be physically overlaying a fraction of another pattern. This kind of interaction manifests itself by an increased number of topological outliers and has been in focus within all previous parts of this paper. The second possible way of cross-pattern interaction is a cross-wise mutual consideration of points without physical overlap. This refers to the consideration of observations from one pattern, while having adjusted the focus of an analysis to that of another involved pattern. This type of misleading interaction becomes important when the two sub-patterns possess different statistical characteristics (e.g., mean and variance). In such cases, geometric overlap leads to unrealistic mixture distributions not just within the zones of overlap but also when two patterns are closely neighbored.

We investigate these two situations by proceeding in the following way: We first fix a point pattern at a scale range of [1*m*, 10*m*]. Repetitively, we translate the scale range by one meter and, for each range, create new random patterns. We do not alter the span of the scale ranges because we don’t intend to introduce additional uncontrolled effects. These random patterns are then moved across the surface until an overlapping degree of 23.8% is reached. The term “overlapping degree” thereby refers to two perspectives: We either require 23.8% of the large-scale points to interact with at least one point from their small-scale counterpart (“large-scale perspective”); or adjust the target the other way round (“small-scale perspective”). The value of 23.8% was thereby chosen to stay in accordance with our previous investigations above. The case of true geometric overlap is simulated by moving the patterns until 23.8% of points show increased local eigenvalues. Analogously, mutual consideration is achieved by optimizing the counts of interactions regardless of the eigenvalues. With increasing scale differences, however, the target is not always reachable. In such cases we rather search for the closest solution. Overall, we generated 9,000 patterns of this kind, 100 per scale difference. All results below are based on averaging over these.

**Fig 10:**
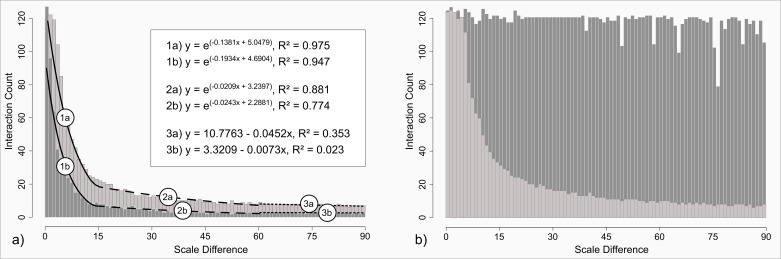
Numbers of interactions between two overlapping patterns across a range of scale differences. a) Overlapping patterns; b) Mutual involvement. Dark gray: small-scale perspective; light gray: large-scale perspective. (1a/b) to (3a/b): fitted decay functions for sub-ranges.

Fig 10a describes the numbers of interactions for the case of a true geometric overlap. Both, small as well as large-scale points do mutually interact in a similar way across scale differences. The only notable difference between them is a differing intensity and can be explained by the generally higher number of points per area for the small-scale process. That is, points from large-scale patterns (the vertical bars in the background) have more small-scale neighbors in their vicinity than vice versa. This increases the general count level and leads to the observed higher count intensity. The functional decay over scale differences cannot be described by a single function. However, we are able to identify three different regimes. When the patterns’ scales are relatively similar, the decay follows a steep exponential curve. Around 15 distance units of scale difference, this relationship is replaced by another, yet flatter, exponential relationship. This function holds up to roughly 61 distance units, where it slowly vanishes into an almost constant level. The latter transition is not an abrupt one, but rather a slow passing over between the functional relations. The thresholds (i.e., 15 and 61 distance units) were assessed by means of cumulatively fitting the different mentioned functions. The supplementary S3 Fig provides the result of this fitting procedure, which in turn reveals the abovementioned thresholds.

Fig 10b illustrates the numbers of interactions for the case of mutual consideration. Other than with the case of geometric overlap, we observe clear differences between large and small-scale patterns. While the large-scale patterns show similar behavior as within Fig 10a, the small-scale patterns show an almost constant level of interaction counts across scale differences. The few observable fluctuations are merely attributable to the inherent randomness in the pattern generation procedure and the general study design. The constant level is explained as follows: After a certain point (which is reached quickly) only one point from each of the large-scale patterns is left for interaction with a fraction of the neighbored small-scale pattern. The point spacing of the large-scale patterns simply becomes too wide-spread to allow any further interaction. That is, the small-scale process falls completely into one of the gaps between two points of the larger-scale process. Thus, the constancy is resultant to the interaction of one large-scale point with a certain fraction of the small-scale points. This finding is highly relevant, because it demonstrates that, when the patterns’ scales are too different from each other, a single point might govern the entire assessment of spatial structure.

#### Influence of Scale Differences on the Disturbing Moran Scatterplot Components

Intuitively, one might argue that the more cross-pattern interactions are observed, the more influences can be expected when performing spatial analysis on these. The number of cross-pattern interactions at least depends heavily on scale differences (as shown above). We should therefore investigate how the three different components from the Moran scatterplot (red, yellow and blue) behave across increasing scale differences between the involved patterns. For that purpose we again use the same 9,000 random patterns as in the previous section. However, this time we additionally assign them Gaussian attributes. These attribute values are drawn from the two Gaussians described in Section ‘Datasets.’ Finally, for each pattern, we calculate the trend lines for the three components and observe their slope over increasingly different scales of the involved overlapping patterns. Thereby, we also vary the direction of the attribute patterns (i.e., increasing from inside to outside vs. decreasing from inside to outside).

**Fig 11:**
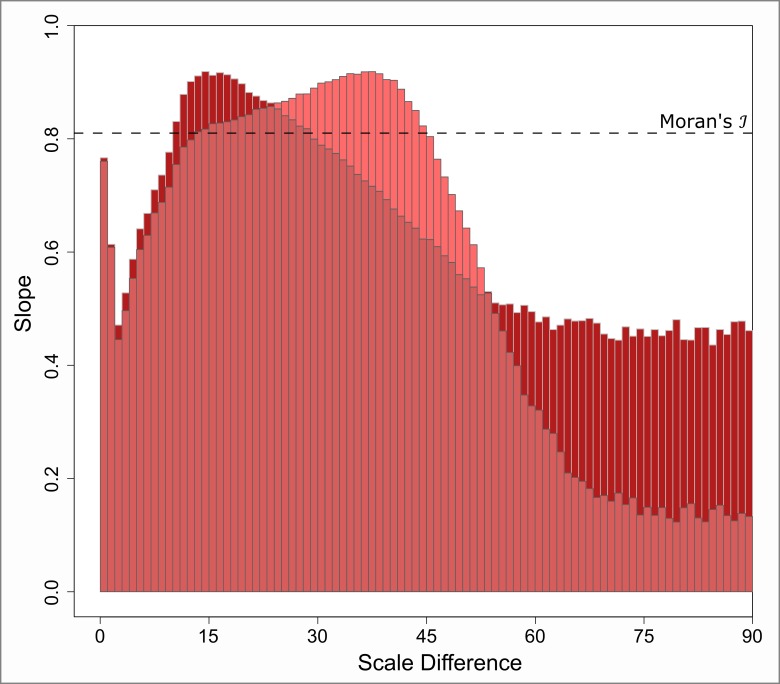
Course of the slope of the red component from the Moran scatterplot. Dark-red: increasing attribute values from pattern center toward the boundary. Light-red: reversed attribute dispersal. The dashed line indicates the true Moran’s ℐ value of 0.81.

Fig 11 shows two characteristic plots obtained for the red component. Recall that this component reflects the non-overlapping parts of the involved patterns from outside the zone of overlap. The dark-red diagram is based on attribute values that increase from center to boundary. Contrary, the light-red diagram reflects reversed attribute dispersal. Two differences are notable: The dark-red plot shows a narrow principal peak, and a slow decay. In contrast, the light-red plot possesses a broader saddle, and then decreases more steeply. The small increase in the very beginning, however, is a commonality shared by both plots.

Now, in order to evaluate meaning and significance of these effects, keep in mind that the actual slope of the red component is 0.81 in case of no overlap. Thus, according to Fig 11, we can figure out two types of configurations under which the slope is quite close to that target. One of these is located at the short-range scale differences where the two patterns are almost similarly scaled (the small peak). Here, the patterns’ interaction is marginal and cross-pattern effects are mostly caused by small fractions of the two boundary regions overlapping each other. However, there are still many points left without any cross-pattern interaction. This preserves the characteristic spatial pattern of the attribute to a large extent, and leads to an almost uninterrupted red component.

A second favorable configuration is observed when the small-scale pattern covers a fraction of the large-scale pattern in a way such that the overall characteristic distribution of attribute values is preserved. In a radial pattern like it is used here, this is the case whenever the small-scale pattern cuts through the large-scale counterpart in a cross-sectional way. However, when the attribute values are dispersed in different ways, the optimal cut-through might appear in a different fashion. Anyway, the consequence of such overlaps is that the red component is not significantly changed in nature. The left-over non-overlapping points do still possess the characteristic distribution of values and, to a large extent, are able to generate a stable red component. Within Fig 11, this configuration is reflected by the two saddles at medium scale differences. The slight differences between light and dark-red within Fig 11 fall back to the type of pattern possessed within the attributes. Thereby, the way of attribute dispersal within the small-scale pattern governs the width of the saddle at the medium scale differences. In contrast, the attribute dispersal of the large-scale pattern is responsible for the steepness and ultimate level of the decay at larger scale differences.

**Fig 12:**
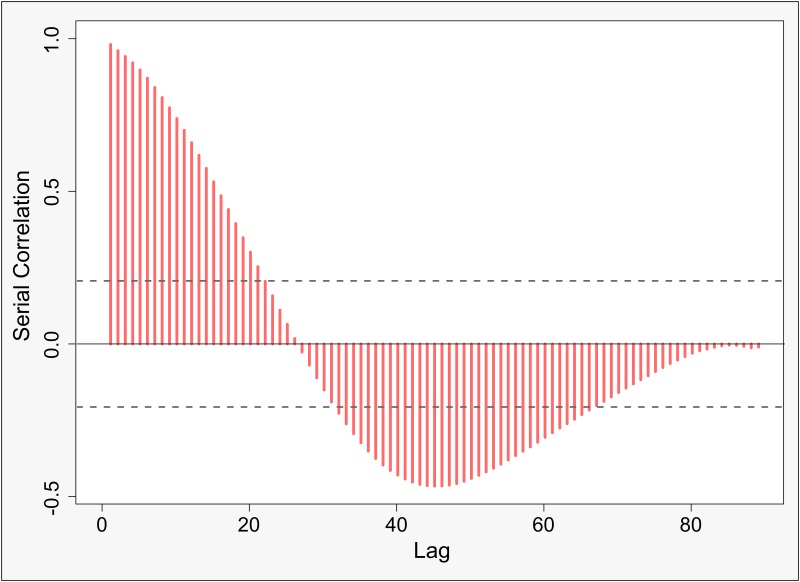
Correlogram of the serial correlation at different lags for the slopes of the red component. Dashed line indicates the 95% confidence interval.

Investigating the serial correlation within the Moran-scatterplot-related slopes of the red component across the scale differences reveals very systematic behavior. The estimated correlogram within Fig 12 shows all possible lags across the whole range of scale differences. It appears to be distinctively smooth, whereas bumps and high frequency fluctuations are not observed. It further indicates two regions in which the autocorrelation between nearby scale differences is significant at the 95% significance level: small lags and medium lags. Thus, as a conclusion, we observe a smooth transition and a slightly sinusoidal seasonality over the sale differences.

**Fig 13:**
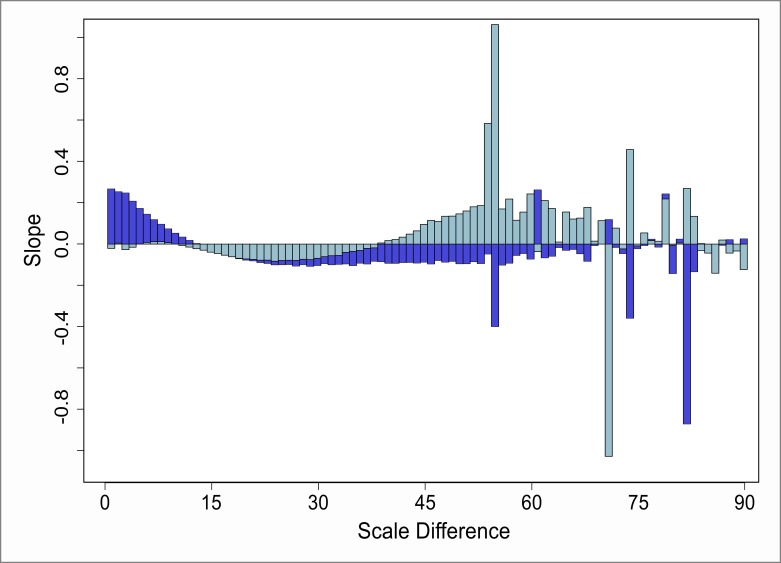
Course of the slope of the blue component from the Moran scatterplot. Dark-blue: increasing attribute values from pattern center toward the boundary. Light-blue: reversed attribute dispersal.

The blue component reflects disturbances which are added by overlapping points originating from the small-scale process. Consequently, Fig 13 shows that the small-scale process itself is the main driver of the shape of the slopes across the scale differences. When the pattern of the attribute values increases from center towards boundary (dark-blue), the component appears slightly positive as long as the involved scales are relatively similar. As the scale differences grow larger, the component transitions into a moderate negative behavior. At a certain point, the pattern becomes chaotic and less predictable. When the direction of the attribute pattern is reversed (light-blue), the course described above is also reversed at larger scale-differences. However, when the scale ranges are more similar, the component tends towards being negative.

**Fig 14:**
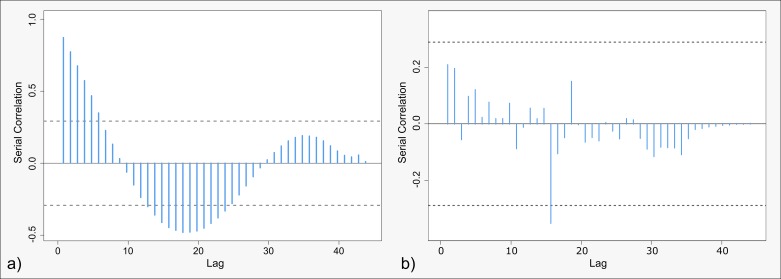
Correlograms of the serial correlation at different lags within the slopes of the blue component. a) Scale differences up to 45 m. b) Scale differences between 45 and 90 m. Dashed line indicates the 95% confidence interval.

The chaotic behavior at larger differences is caused by interaction between few points. The small-scale pattern interacts with only one or two points from the large-scale pattern at these scale differences. Moreover, these few points do in turn interact with large parts of the small-scale pattern. Thus, if the attribute values of these few points are somewhat extreme, a large number of either highly positively or negatively correlated comparisons are included. The quintessence is that, as off a scale distance of approximately 45 meters, the way how the patterns interact is no longer predictable with respect to the blue component. The explained chaotic behavior is also well reflected by the serial correlation given by Fig 14. Thereby, unlike with the red component above, we separated the correlogram into two parts. The first of these demonstrates a coherent behavior up to scale differences of 45 m. Here, the autocorrelation progresses smoothly. The second one reflects the chaotic behavior at larger differences. Clearly, the high level of fluctuation barely allows any indication for systematic behavior. However, Fig 13 indicates that there is a slight tendency towards either positive or negative slopes for each of the two investigated attribute patterns. That is, this tendency seems to flip when the pattern gets reversed. Further, note the similarity of the serial correlation at small scale differences and that of the red component. This indicates that the blue component has a strong influence on small scale differences.

**Fig 15:**
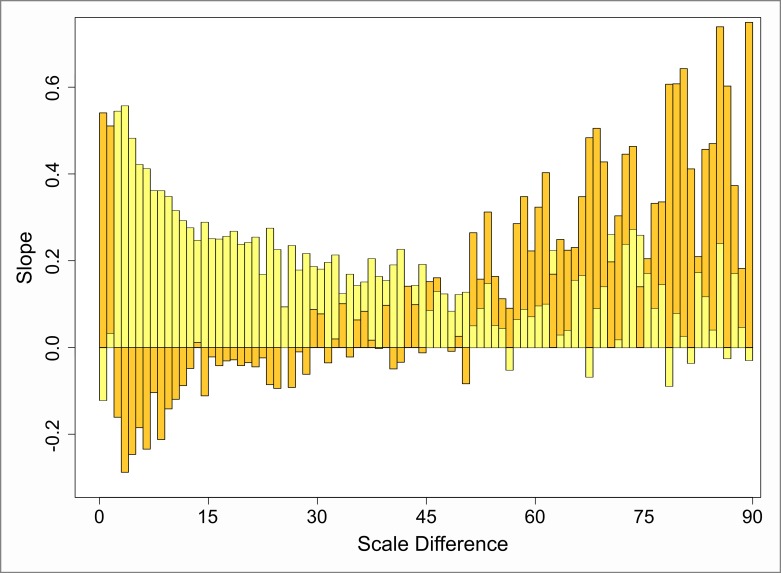
Course of the slope of the blue component from the Moran scatterplot. Light-yellow: increasing attribute values from pattern center toward the boundary. Dark-yellow: reversed attribute dispersal.

In analogy to the small-scale pattern with the blue component, the large-scale pattern is the main driver of the yellow component (Fig 15). When the attribute pattern increases from center to boundary, the yellow component is positively dominant at small scale differences (light-yellow). When the pattern is reversed, however, this relationship is flipped and the yellow component becomes dominant at larger differences (dark-yellow). Interestingly, the role of the small-scale process here is to control the direction of the component. When the small-scale attribute pattern runs opposite to the larger-scale one, the yellow component is turned to negative either at small or large scale differences.

**Fig 16:**
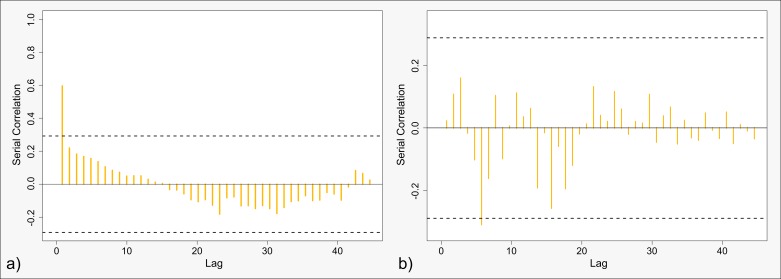
Correlograms of the serial correlation at different lags within the slopes of the yellow component. a) Scale differences up to 45 m. b) Scale differences between 45 and 90 m. Dashed line indicates the 95% confidence interval.

The serial correlation of the yellow component reveals a similar smoothness as with the blue component. However, as none of the serial correlations is significant, this component is more volatile and less coherent than the blue counterpart. Notice the isolated spike at small lags in Fig 16a. This isolated spike indicates that the pattern does not possess abrupt bumps, because neighboring values are to a certain extent similar. However, this similarity decreases quickly. Again, the clutter increases strongly for the larger scale differences. Just like with the blue component, this unveils a two-pattern regime (16a vs. 16b).

## Discussion

The tweets from London used in our study have shown clear indications of geometrically overlapping phenomena and processes. The derived semivariogram shows unusual behavior and hints on repulsion, and thus negative spatial autocorrelation at local scales. The local-scale activity is not surprising given that urban areas are typically patchy and dense. What is surprising though is the negative (repulsion) behavior. Besides, the peak in the semivariogram is rather low, which indicates merely negligible spatial behavior in the variable (which is not plausible in an urban environment). A closer look at the pairwise autocovariance terms and their associated geographic distances reveals that both clustering and repulsion take place in close vicinity to each other, besides a large amount of unrelated tweets. These results demonstrate that the spatial associations of interest (mostly those of clustering nature) may remain hidden, and spurious spatial relationships might instead be detected. These results strongly support our initial hypothesis of overlapping phenomena being reflected within Twitter datasets. This is, however, ultimately leading to a violation of the requirement of second-order stationarity, whereupon many spatial-statistical techniques are based (and so is Moran’s ℐ, ([56], p.166)).

We analyzed the artificial spatial regime that forms within zones of overlap by means of the eigenvalues of local as well as global spatial weights. This regime is characterized by a large number of topological outliers. These are known from traditional datasets where they occur after applying some kind of normalization procedure [49, 50]. With social media data, however, these outliers also occur without any further modification of the data as a result of overlap. They increase the topological variability, i.e., the overall chances for detecting spurious spatial interaction and patterns. Further, they decrease the power as well as the efficiency of spatial test statistics, which in turn leads to a higher risk for drawing wrong conclusions (i.e., Type I and II errors). We only tested overlapping patterns of roundish shape, which faintly limits the results to these. However, other kinds of patterns should, by principle, behave in a similar way.

These topological outliers have impact on the detection of spatial structure by adding different kinds of disturbances. These manifest themselves in two different ways: One disturbing component is related to overlapping observations which belong to the smaller-scale pattern of the two investigated ones. A second component is related to the points of the large-scale pattern respectively. Both show different behaviors, but, however, are inherently linked. Their mutual relationship is demonstrated by their causal mechanisms. These are both driven by interactions between the two involved patterns. Further, both kinds of disturbances correlate with the degree of topological variation, though in different ways. One component might correlate positively, while the other one associates in a negative way. As a result, these components do in fact lower the strength of detectable spatial effects and might lead to misleading interpretations of spatial patterns. Another interpretation of these nuisances from social media characteristics is to see them as distinct spatial processes. The disturbing components come up with their own spatial interaction behavior and disturb the actual pattern of interest. The latter is true because they are not caused by real-world social phenomena, which in turn get obscured by them.

Observing these disturbing components over a range of scale differences between both involved patterns unveils several kinds of effects. First of all, the degree of mutual interaction seems to follow several forms of exponential decay functions as scale differences increase. This decay starts out steep and then transitions into a flatter exponential function before slowly vanishing towards an almost constant level at very large differences. When differently scaled patterns are neighbored instead of overlapping geometrically, small and large-scale patterns react differently. Small-scale patterns tend to interact with few points from the large-scale process. Thus, few points govern the results of a spatial analysis in such cases. When these considered large-scale points are extreme with respect to their attribute, any result might be strongly biased. In contrast, the large-scale pattern, again, shows an exponential decay like described above.

In terms of the direction of the components (i.e., adding negative or positive spatial autocorrelation to the Moran scatterplot), the achieved results provide a diverse picture. The red component (consisting of non-overlapping observations) should either overlap in a way such that only smaller parts of the boundaries of the patterns interact with each other. Another low risk option is an overlap that cuts through the attribute values of the larger-scale pattern so that all characteristic parts of the disturbed pattern are retained in accordance to their proportion within that pattern. In all other cases, however, the characteristics of large-scale patterns get disturbed significantly, and results become increasingly unrealistic. However, since we analyze positively autocorrelated data, the red component remains positive across all tested scale-differences.

The blue and yellow disturbances (i.e., those caused by either the smaller or the larger-scale process) behave in more complex ways. These processes are strongly dependent of the actual pattern of the attribute value dispersal. However, as a summarizing result, these components do typically provide ranges in the scale differences at which they add negative influences. Similarly, at some other subranges, these relationships turn toward positive respectively. Further, as the scale differences between overlapping patterns become larger, these components show increasingly chaotic, and thus unpredictable behavior. The latter effect is caused by interactions between only few points from a larger-scale pattern with many of those from a smaller-scale opponent.

Our results reveal some limitations of our research. First of all, we did not remove artifacts like bot-produced tweets from the data. These might contribute content of little explanatory power with respect to real-world social phenomena (see [57] for their impact on altmetrics). Therefore, it remains unknown to what extent these tweets play a role in spatial patterning. Further, we investigated a limited number of types of spatial attribute configurations (radial, increasing attributes from inside towards the borders and vice versa) and narrowed down the scope to an overlap of only two patterns. Apart from topological considerations, we also held statistical properties like the means and variances of the attribute patterns constant across our investigations. The reason for both these choices was to keep the analyses tractable and to facilitate their interpretation, but they might play a role in the results. Moreover, we exclusively focused on positive spatial autocorrelation given its higher practical relevance. Nevertheless, findings about negatively correlated patterns under heterogeneous conditions caused by overlaps would be of interest for the study of spatial outliers. From a technological perspective, we restricted our analysis to explicit coordinates by leaving out coordinates obtained through geocoding.

## Conclusions

Social media data reflect an ample amount of social phenomena and processes. These are likely to appear overlapping in space and time and are prone to varying interpretations among the contributing users. In this paper we investigate how topological effects caused by these overlaps influence outcomes of spatial analyses. For that purpose, we first analyzed the spatial behavior of LDA-derived semantic topic associations within a Twitter sample from London. Afterwards, we conducted a number of simulation experiments to investigate different aspects related to the topology of overlapping point patterns. We enriched these simulated patterns by Gaussian attribute values at different means but with similar variance. They thus resemble a special case of spatial heterogeneity in which different regimes are not just appearing close to each other (the traditional notion), but form an artificial regime in-between through geometric overlap.

To summarize our results we list the key findings in the following enumeration. These points are also meant to raise scholars’ awareness of carefully undertaking spatial analyses of social media data:

- Increased numbers of topological outliers are found and these increase the risk of false positives and negatives in spatial analyses on social media data. Thus, misleading indications regarding spatial relationships within the data must be expected when using established spatial analysis methods.
- The way how spatial proximity is modelled through spatial weight matrices is crucially important in general, but even more so with overlapping patterns. The tested configurations have shown a large variety and thus sensitivity to this issue. Distance-based weights are extremely problematic on that regard, since they possess extreme behavior at short distances. The latter happens frequently when patterns overlap.
- When differently scaled patterns overlap and when the scale differences are large, single extremal points from the larger-scaled of the involved patterns might control the results significantly.
- When social media patterns are geometrically overlapping, the number of interactions, and thus the chance for detecting spurious effects, decreases exponentially with increasing scale differences. In contrast, when differently scaled patterns are just neighbored in close vicinity, the adjusted analysis scale becomes important for the risk of including wrong observations.
- Besides scales of the involved patterns, the shape of how the attribute values are dispersed possesses great influence on the type of interferences. These might either be expressed in terms of an additional positive or a negative component respectively. The latter act like additional spatial processes that interfere with the actual pattern of interest.

Future research should focus on a range of different aspects that could not be investigated exhaustively within this paper. One of these is the pattern of the attribute values. We used two different kinds of radially dispersed trends within each of our point patterns. However, different kinds of attribute value arrangements might lead to different results. Our tests have shown respective indications for a tremendous sensitivity to this issue. Further, our results indicate interaction with the kind of neighborhood definition. We used distance-based spatial weights and roughly tested some binary configurations. These experiments, however, unveiled a high variation among different types of weights. This is an issue of high practical relevance and thus deserves particular attention. Further, given their practical relevance and widespread use, the severe behavior of distance-based weights at short distances should be further examined with respect to social media data as they are commonly used. This should incorporate a critical discussion of results achieved through already conducted spatial studies of social media. Other future prospects might include variations of statistical properties such as means and variances as well as including coordinates from geocoded text-based information from the posted messages.

We close the article with some recommendations to researchers conducting spatial analysis with georeferenced social media feeds. In the first place, one should check the data for signs of heterogeneities. This should incorporate the geometric dimension (e.g., through techniques like Ripley’s K function [58]) as well as the respective attribute (techniques like local spatial heteroscedasticity (LOSH) might be helpful [59, 60]). As we have seen with the semivariogram in our analysis, one difficulty is that the heterogeneity might remain hidden because of the noisy nature of the data. Therefore, whenever possible, the target set of observations shall be isolated as far as possible from the rest. Clearly, this is hampered by the oftentimes exploratory character of spatial analysis. Spatial patterns are often part of an early investigation in the hypothesis building phase when the dataset is not well-understood. This requires the acquisition of extensive expert knowledge about the spatial aspects of the target subject of investigation. This leads to the next recommendation which is putting vast effort in properly designing the spatial weights. This is an essential part of each spatial analysis. However, our eigenvalues analysis has shown that it is even more important when it comes to social media. The spatial weights matrix needs to be constrained to the particular research needs as conservatively as possible. In the aftermath when it comes to drawn inference, a double-check needs to be performed whether the reference distribution under the null hypothesis is really appropriate. Again, the outliers might lead to an unexpected shape of this distribution, which would ultimately lead the analyst to wrong conclusions.

## Supporting Information

S1 Dataset Twitter sample. See the respective attached file.
(ZIP)

S2 Dataset Simulated data used in Sections ‘Influences on Spatial Autocorrelation’ and ‘Increased Topological Variability.’ See the respective attached file.
(ZIP)

S3 Dataset Simulated data (“inclusion” and “large-scale perspective”) used in Section ‘Influence of Scale Differences on the Numbers of Interactions.’ See the respective attached file.
(ZIP)

S4 Dataset Simulated data (“inclusion” and “small-scale perspective”) used in Section ‘Influence of Scale Differences on the Numbers of Interactions.’ See the respective attached file.
(ZIP)

S5 Dataset Simulated data (“overlap” and “large-scale perspective”) used in Section ‘Influence of Scale Differences on the Numbers of Interactions.’ See the respective attached file.
(ZIP)

S6 Dataset Simulated data (“overlap” and “small-scale perspective”) used in Section ‘Influence of Scale Differences on the Numbers of Interactions.’ See the respective attached file.
(ZIP)

S1 Fig Spatial distribution of the Gaussian attribute values across the single pattern (top) and their histogram (bottom). (TIFF)

S2 Fig Spatial distribution of the Gaussian mixture across a combined pattern (top) and their joint histogram (bottom). (TIFF)

S3 Fig Goodness of fit for the fitted functions. Blue: exponential function; red: linear function. Please read the fits in a cumulative way. The exponential function was evaluated from left to right. That is, the determined optimum at 15 means that the first 15 meters of the course follow the respective function. In contrast, the red linear function needs to be read in a reversed order. The trailing scale differences as off 61 m proceed like the fitted function.
(TIFF)

S4 Fig Heat map of pairwise covariance terms and semivariogram of topic associations. The white semivariogram plotted atop of the heat map refers to the right-hand y-axis. The left-hand y-axis is associated with the underlying color-coded bins of the heat map. This figure is similar to Fig 3, but shows relative heat map values for reasons of comparison (i.e., heat map values are normalized by rows). Part a) contains all tweets while part b) is adjusted for spatial coincident tweets.
(TIFF)
