# Correction: Abundant Topological Outliers in Social Media Data and Their Effect on Spatial Analysis

**DOI:** 10.1371/journal.pone.0209722

**Published:** 2018-12-19

**Authors:** Rene Westerholt, Enrico Steiger, Bernd Resch, Alexander Zipf

The following information is missing from the Funding section: We would like to express our gratitude to the Austrian Science Fund (FWF) for supporting the project “The Scales and Structures of Intra-Urban Spaces” (reference number P29135-N29).
