# Supplementary material for: Abundant Topological Outliers in Social Media Data and Their Effect on Spatial Analysis
Source: PLoS One. 2016 Sep 9;11(9):e0162360. doi: 10.1371/journal.pone.0162360 (PMC5017681; doi:10.1371/journal.pone.0162360)
Supplement: S1 Dataset — See the respective attached file. (ZIP) [file pone.0162360.s001.zip › Readme_Twitter_Data.docx]

**S1 Data Description: Twitter Sample from London**

| Quick Facts |  |
| --- | --- |
| Bounding Box | xMin: 696345.13; yMin : 5704812.23; xMax: 710008.15; yMax : 5715956.11. |
| Number of Tweets | 23.873 |
| Sampling Period | 15 October 2013 – 28 July 2014 |
| Geographic Projection of the Coordinates | EPSG: 32630 (UTM Zone 30 North) |
| Encoding | UTF-8 |
| Decimal Separator | Point |
| CSV Delimiter | Semicolon |
| Anonymized | Yes |

**Columns:**

**Column 1: X**

This column contains the X part of the coordinate. Data type is *double*.

**Column 2: Y**

This column contains the Y part of the coordinate. Data type is *double*.

**Column 3: TWEETCONTE**

This column contains the posted text of the tweet. Data type is *String*.

**Column 4: TOPIC_INDI**

This column contains the topic association calculated after modelling topic through Latent Dirichlet Allocation (see Section ‘Datasets’). Data type is *double*, unit is *fraction of 100*.

**Column 5: TIMESTAMP**

This column represents the timestamp at which the tweet was posted. Data type is *timestamp*. We adhere to ISO 8601 by using the standard format *YYYY-MM-DDTHH:MM:SS+00:00*, whereby the trailing additive time shift of 00:00 indicates UTC.
