# Supplementary material for: Abundant Topological Outliers in Social Media Data and Their Effect on Spatial Analysis
Source: PLoS One. 2016 Sep 9;11(9):e0162360. doi: 10.1371/journal.pone.0162360 (PMC5017681; doi:10.1371/journal.pone.0162360)
Supplement: S2 Dataset — See the respective attached file. (ZIP) [file pone.0162360.s002.zip › Readme_Overlapping_Pattern.docx]

**S2 Data Description: Simulated Overlapping Pattern**

| Quick Facts |  |
| --- | --- |
| Bounding Box | xMin: 3564.71; yMin : 2265.23; xMax: 6555.89; yMax: 4513.21. |
| Number of Observations | 1.000 |
| Sampling Period | N/A |
| Geographic Projection of the Coordinates | EPSG: 3857 (Pseudo Mercator) |
| Encoding | UTF-8 |
| Decimal Separator | Point |
| CSV Delimiter | Semicolon |
| Anonymized | N/A |

**Columns:**

**Column 1: ID**

This column contains unique IDs of the observations. Data type is *integer*.

**Column 2: X**

This column contains the X part of the coordinate. Data type is *double*.

**Column 3: Y**

This column contains the Y part of the coordinate. Data type is *double*.

**Column 4: CLUST**

This column contains the assignment to one of the two overlapping clusters. Data type is *integer*. 1 indicates association to the small-scale cluster, 2 indicates association to the large-scale cluster.

**Column 5: S_CLOSE_L**

This column contains an indicator that signals whether a small-scale observation interacts with a large-scale observation (1) or vice versa (2). If an observation is not interacting spuriously the value is 0. Data type is *integer*.

**Column 6: LOCEIG50**

This column contains the local eigenvalues of the spatial weights matrix (IDW) adjusted to the small-scale pattern. Data type is *double*.

**Column 7: LOCEIG80**

This column contains the local eigenvalues of the spatial weights matrix (IDW) adjusted to the large-scale pattern. Data type is *double*.

**Column 8: LOCEIG50_C**

This column contains the local eigenvalues of the C-coded spatial weights matrix (IDW) adjusted to the small-scale pattern. Data type is *double*.

**Column 9: LOCEIG50_W**

This column contains the local eigenvalues of the W-coded spatial weights matrix (IDW) adjusted to the small-scale pattern. Data type is *double*.

**Column 10: LOCEIG80_W**

This column contains the local eigenvalues of the W-coded spatial weights matrix (IDW) adjusted to the large-scale pattern. Data type is *double*.

**Column 11: LOCEIG80_C**

This column contains the local eigenvalues of the C-coded spatial weights matrix (IDW) adjusted to the large-scale pattern. Data type is *double*.

**Column 12: ATTRIBUTE**

This column contains the Gaussian attribute values. Values are drawn from two Gaussians: N(250, 150) and N(750, 150). Data type is *double*.

**Column 13: ATTRIBUTE_STD**

This column contains the standardized attribute values (i.e., $ATTRIBUTE_{STD}=\frac{ATTRIBUTE-\bar{ATTRIBUTE}}{s_{ATTRIBUTE}}$). Data type is *double*.

**Column 14: LAG50**

This column contains the spatial lag after applying the spatial weights matrix of the small-scale pattern to the respective standardized attribute values from Column 13. Data type is *double*.

**Column 15: LAG80**

This column contains the spatial lag after applying the spatial weights matrix of the large-scale pattern to the respective standardized attribute values from Column 13. Data type is *double*.
