# Supplementary material for: Abundant Topological Outliers in Social Media Data and Their Effect on Spatial Analysis
Source: PLoS One. 2016 Sep 9;11(9):e0162360. doi: 10.1371/journal.pone.0162360 (PMC5017681; doi:10.1371/journal.pone.0162360)
Supplement: S3 Dataset — See the respective attached file. (ZIP) [file pone.0162360.s003.zip › Readme_Inclusion.docx]

**S3 + S4 Data Description: Inclusion**

This folder contains two subfolders. One of these is entitled by “inclusion_lws.” It contains the data used for calculating the numbers of interactions across different patterns for the case of mutual inclusion (see paper). “lws” thereby is an acronym for “large with small,” and denotes the perspective from which the inclusion was performed. “swl” stands for “small with large” respectively.

Please find more information about the two respective data sources in additional readme files placed within the subfolders.
