# Supplementary material for: Abundant Topological Outliers in Social Media Data and Their Effect on Spatial Analysis
Source: PLoS One. 2016 Sep 9;11(9):e0162360. doi: 10.1371/journal.pone.0162360 (PMC5017681; doi:10.1371/journal.pone.0162360)
Supplement: S6 Dataset — See the respective attached file. (ZIP) [file pone.0162360.s006.zip › S6_overlap_swl/Readme_Overlap_SWL.docx]

**S9 Data Description: Overlap_SWL**

| Quick Facts |  |
| --- | --- |
| Total Number of Datasets | 91 |
| Number of Observations per Dataset | 1.000 |
| Sampling Period | N/A |
| Geographic Projection of the Coordinates | EPSG: 3857 (Pseudo Mercator) |
| Encoding | UTF-8 |
| Decimal Separator | Point |
| CSV Delimiter | Semicolon |
| Anonymized | N/A |

**File “numbers_of_included.csv”**

This file contains the numbers of cross-pattern interactions across all tested scale differences. The order is ascending, starting at scale-range [2,11].

**Columns:**

**Column 1**

This column contains the numbers of cross-pattern interactions. Data type is *integer*.

**Files “clust_2_11.csv” – “clust_91_100.csv”**

These files contain the patterns which were used for deriving the numbers from file “numbers_of_included.csv.” The numbers contained in the file names denote the respective spatial scale ranges at which the associated points interact. Note that with each of these files, the first 500 rows contain the small-scale pattern against which the larger-scale ones were compared. This small-scale pattern does interact at [1,10] in any case. This is followed by the trailing 500 lines which represent the respective opponent.

**Columns:**

**Column 1**

This column contains the X part of the coordinate. Data type is *double*.

**Column 2**

This column contains the Y part of the coordinate. Data type is *double*.

**Column 3**

This column indicates membership of a point to one of the two sub-patterns. The number 0 thereby indicates membership within the small-scale pattern. Any other number indicates membership of the respective second sub-pattern contained. Data type is *integer*.
