# Supplementary figures and images for: Abundant Topological Outliers in Social Media Data and Their Effect on Spatial Analysis
Source: PLoS One. 2016 Sep 9;11(9):e0162360. doi: 10.1371/journal.pone.0162360 (PMC5017681; doi:10.1371/journal.pone.0162360)

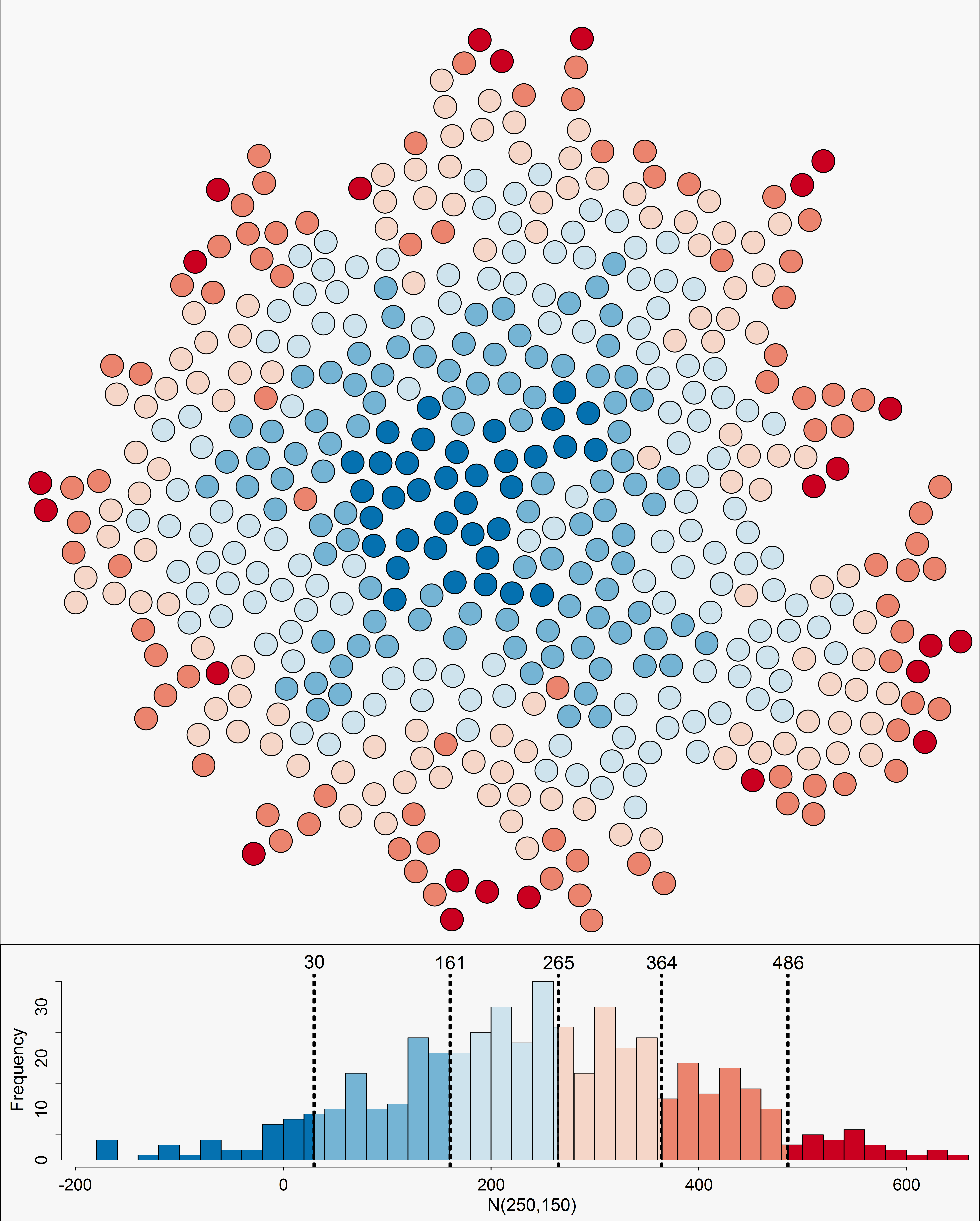

Supplement: S1 Fig — (TIFF) [file pone.0162360.s007.tiff]

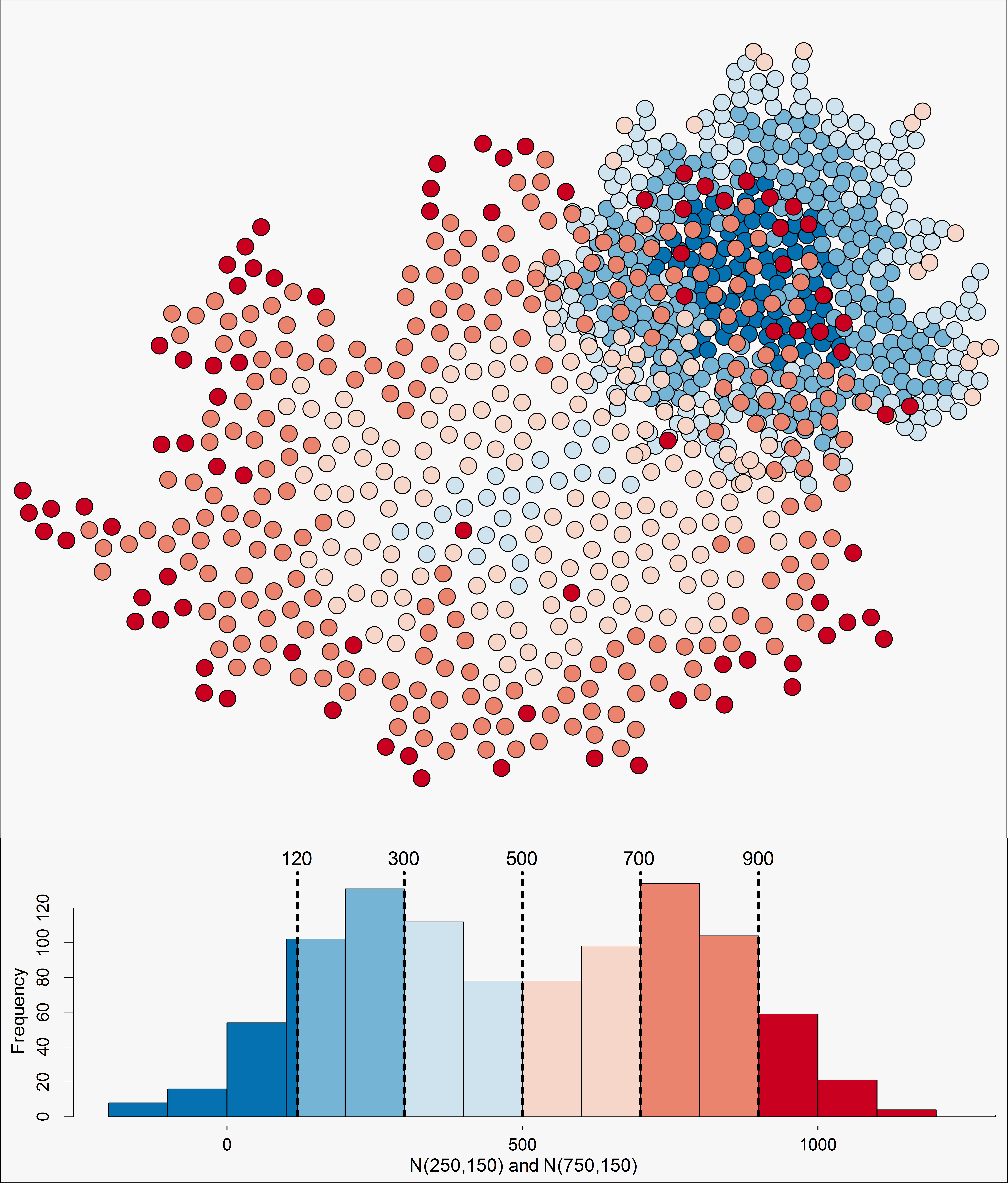

Supplement: S2 Fig — (TIFF) [file pone.0162360.s008.tiff]

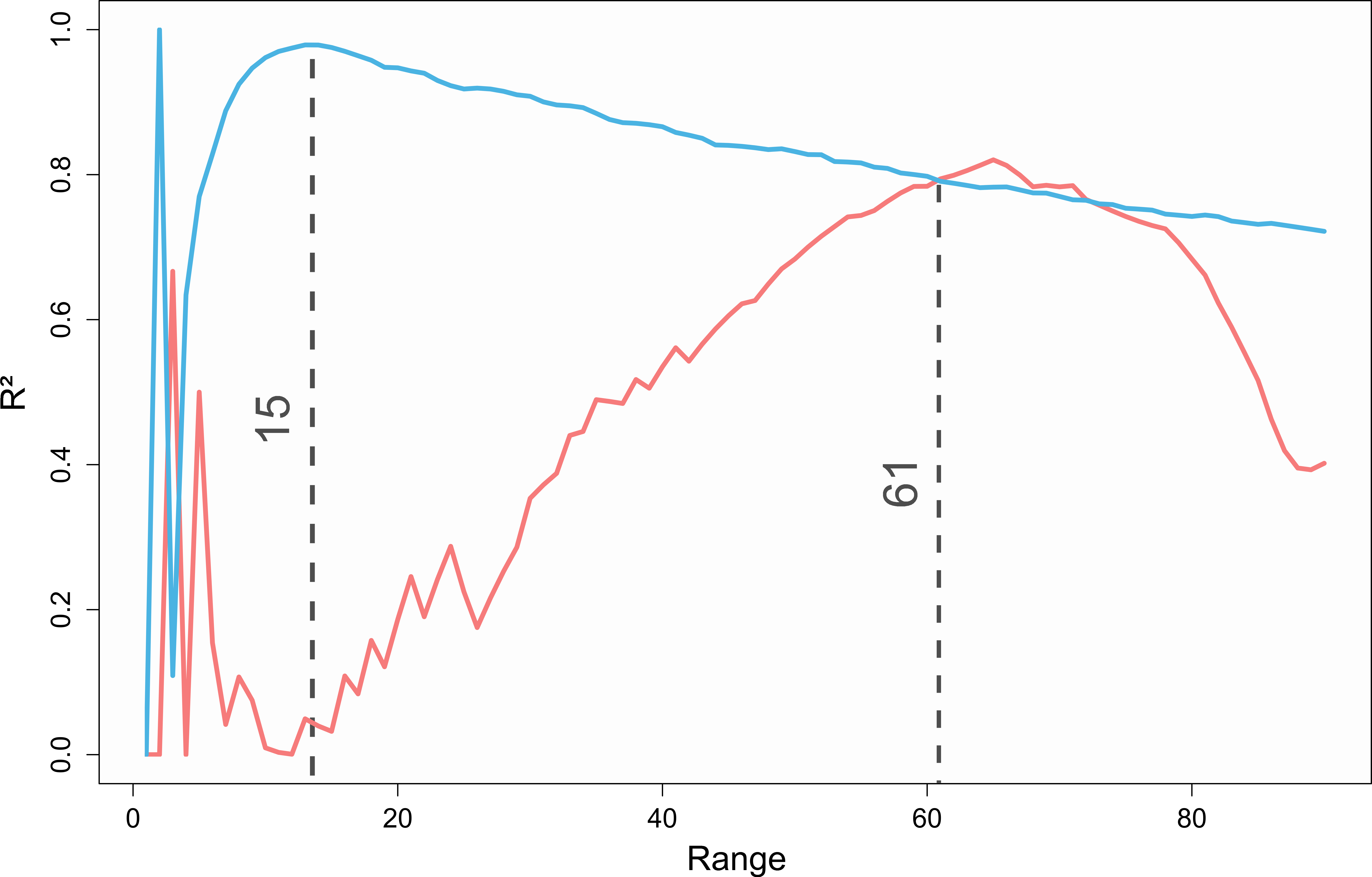

Supplement: S3 Fig — Blue: exponential function; red: linear function. Please read the fits in a cumulative way. The exponential function was evaluated from left to right. That is, the determined optimum at 15 means that the first 15 meters of the course follow the respective function. In contrast, the red linear function needs to be read in a reversed order. The trailing scale differences as off 61 m proceed like the fitted function. (TIFF) [file pone.0162360.s009.tiff]

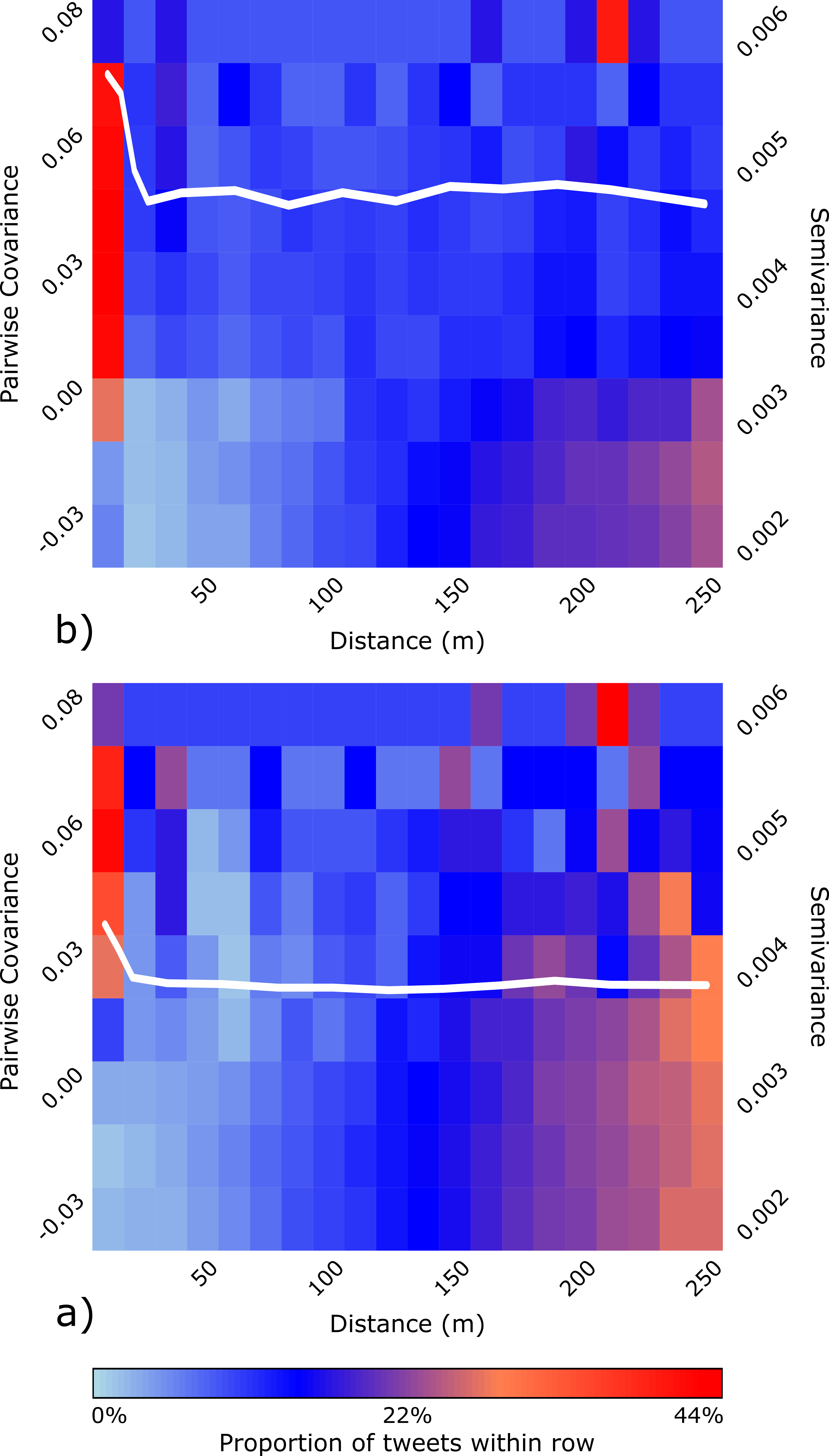

Supplement: S4 Fig — The white semivariogram plotted atop of the heat map refers to the right-hand y-axis. The left-hand y-axis is associated with the underlying color-coded bins of the heat map. This figure is similar to Fig 3, but shows relative heat map values for reasons of comparison (i.e., heat map values are normalized by rows). Part a) contains all tweets while part b) is adjusted for spatial coincident tweets. (TIFF) [file pone.0162360.s010.tiff]
